# Cannabinoid antagonist SLV326 induces convulsive seizures and changes in the interictal EEG in rats

**DOI:** 10.1371/journal.pone.0165363

**Published:** 2017-02-02

**Authors:** Martin F. J. Perescis, Natasja de Bruin, Liesbeth Heijink, Chris Kruse, Lyudmila Vinogradova, Annika Lüttjohann, Gilles van Luijtelaar, Clementina M. van Rijn

**Affiliations:** 1 Donders Institute for Brain, Cognition and Behaviour, Radboud University, Nijmegen, The Netherlands; 2 HAS University of Applied Sciences, ‘s-Hertogenbosch, The Netherlands; 3 Abbott Healthcare Products BV (formerly Solvay Pharmaceuticals), Weesp, The Netherlands; 4 Institute of Higher Nervous Activity and Neurophysiology, Russian Academy of Sciences, Moscow, Russia; 5 Institut für Physiologie I, Westfälische Wilhelms Universität Münster, Münster, Germany; University of Catanzaro, ITALY

## Abstract

Cannabinoid CB1 antagonists have been investigated for possible treatment of e.g. obesity-related disorders. However, clinical application was halted due to their symptoms of anxiety and depression. In addition to these adverse effects, we have shown earlier that chronic treatment with the CB1 antagonist rimonabant may induce EEG-confirmed convulsive seizures. In a regulatory repeat-dose toxicity study violent episodes of “muscle spasms” were observed in Wistar rats, daily dosed with the CB1 receptor antagonist SLV326 during 5 months. The aim of the present follow-up study was to investigate whether these violent movements were of an epileptic origin. In selected SLV326-treated and control animals, EEG and behavior were monitored for 24 hours. 25% of SLV326 treated animals showed 1 to 21 EEG-confirmed generalized convulsive seizures, whereas controls were seizure-free. The behavioral seizures were typical for a limbic origin. Moreover, interictal spikes were found in 38% of treated animals. The frequency spectrum of the interictal EEG of the treated rats showed a lower theta peak frequency, as well as lower gamma power compared to the controls. These frequency changes were state-dependent: they were only found during high locomotor activity. It is concluded that long term blockade of the endogenous cannabinoid system can provoke limbic seizures in otherwise healthy rats. Additionally, SLV326 alters the frequency spectrum of the EEG when rats are highly active, suggesting effects on complex behavior and cognition.

## Introduction

Cannabinoid antagonists have been of interest because of their high therapeutic potential for a wide range of ailments, including addiction, obesity and metabolic disorders [1]. However, long-term use of these drugs might compromise psychiatric safety. Rimonabant (SR141716A, Acomplia®, Zimulti®), which has been licensed for the treatment of overweight adult patients [2], was withdrawn from the market in 2009 because it might facilitate depressive disorders [3]. Around the same time, the development of other CB1 antagonists and inverse agonists was discontinued. However, the continuation of research on cannabinoid antagonists remains relevant, because the endocannabinoid system is involved in controlling homeostasis of many systems, and therefore regulation of an overactive endocannabinoid system might have beneficial effects on a wide variety of conditions [4].

Clinical reviews on safety and efficacy stated that rimonabant should be used with caution in epilepsy patients [5], since there were reports in both mice and humans that rimonabant might induce epilepsy. Indeed, our study in healthy Wistar rats, which were neither prone to epilepsy nor had spontaneous seizures of any type, showed the occurrence of severe convulsive seizures after a few daily doses of this cannabinoid antagonist [6]. There is also some evidence in humans: partial seizures were observed in a patient treated with rimonabant for obesity, and with a history of idiopathic generalized epilepsy [7]. Katona and Freund [8] already mentioned that cannabinoid antagonists may hold risks in individuals with a history of convulsive epilepsy.

In addition, rimonabant induced status epilepticus-like activity in a neuronal cell culture model of acquired epilepsy [9], whereas the agonist of cannabinoid receptor type 1 (CB1), WIN55,212–2 reduces status epilepticus and subsequent mortality in rats [10]. CB1 agonists attenuate several types of epileptiform seizures [11–13], whereas antagonists counteract these effects. Agonists also retard the development of kindling [14, 15]. In the pilocarpine rat model for epileptogenesis, cannabinoid agonists abolished the occurrence of late spontaneous epileptic seizures, whereas rimonabant increased both the seizure frequency and duration [16]. Mutant mice, lacking CB1 receptors in the hippocampus, are more vulnerable to kainic acid-induced seizures than their wild-type counterparts [17, 18], while endocannabinoid enhancement protected against kainic acid-induced seizures [19].

Consequently, both in vivo and in vitro data suggest that antagonizing the cannabinoid system increases the risk of developing epilepsy. It is hypothesized that de novo seizures in non-epileptic healthy subjects may arise when their endocannabinoid system is blocked because it plays a crucial role in protecting the brain from seizures [6]. All the seizures observed in rats exposed to rimonabant were of limbic origin. Indeed, although the endocannabinoid system’s most abundant receptor, CB1, can be found throughout the central nervous system, it is present in high density in the amygdala, prefrontal cortex, and hippocampus [20]. However, due to the wide distribution of CB1 receptors throughout the CNS, it is highly unlikely that chronic blocking of the CB1 receptor is only manifested in seizure activity. And indeed, mice treated with CB1 agonist WIN 55–212,2 during adolescence display a permanent suppression of pharmacologically induced cortical oscillations [21]. The effects of blocking the CB1 receptor on cortical oscillations, however, were not investigated.

Until now, seizure activity has not been reported for cannabinoid antagonists other than rimonabant. During a 5-month regulatory required repeated dose toxicity study for possible adverse effects of chronic treatment with the CB1 receptor antagonist SLV326 in adult Wistar rats, violent, highly stereotypical episodes of muscle spasms were observed during daily clinical observations. Although these observations were restricted to the regular working hours, it became evident that a high numbers of animals (70% of the animals from the SLV326-treated group; personal communication) were affected. A subgroup of these animals was selected for an EEG study, with the objective to investigate these potential seizures as well as the interictal EEG.

Seizures can occur irregularly and might therefore stay undetected in a relatively short (24 hour) EEG study. Therefore, interictal spikes were quantified. Interictal spikes can serve as a sensitive marker for epilepsy [22], even when the relatively rare, and therefore more difficult to catch, seizures are not (yet) found.

It is highly unlikely that chronic blocking of the CB1 receptor is only manifested in seizures. CB1 agonists have been described to alter the EEG frequency spectrum [23, 24]. No such data are available, however for CB antagonists. Therefore, the spectral content of the ongoing EEG were observed during the study.

## Methods

The study was performed in accordance with the guidelines of the European Community for the use of experimental animals and was approved by the Radboud University Institutional Animal Care and Use Committee (IACUC) (RUDEC-2007-161).

### Animals

For this study 35 Crl:WI Wistar rats (Charles River Laboratories, Sulzfeld, Germany), aged 7 months, were used, 17 male and 18 female animals. The rats were selected from a group of 100 male and 100 female, belonging to a regulatory required repeated dose toxicity study with SLV326. Following the observations of violent muscle movements and convulsions, the regulatory study was stopped and 35 rats were transferred to Radboud University for further investigations. In total, 24 animals (12 M, 12F) had received SLV326, and 11 animals (5M, 6F) had received vehicle only and served as control group. In the SLV326 group, 50% of the 24 animals had a history of at least 1 documented episode of violent muscle movements.

During the regulatory study, the animals were housed under Specified Pathogen Free conditions, 5 animals of the same sex per cage, with food and water ad libitum. To ensure that the rats, nocturnal animals by nature, were at their most active during the day, the storage and experiment rooms had a reversed, 12–12 light-dark cycle with lights on at 8:00 a.m.

At Radboud University, Nijmegen, the animals were housed individually with food and water ad libitum. Envirodry served as cage enrichment. Storage room as well as recording room had a reversed 12–12 light-dark cycle with lights off at 8:00 a.m.

After completion of the experiments, the animals were euthanized by means of a lethal dose of pentobarbital, injected intraperitoneally.

### Drug

From an age of 8–9 weeks, SLV326 was administered daily by oral gavage. The drug was dissolved in a semi-solid solution with Cremophor. The male rats received 3 mg/kg, the females 2 mg/kg. Administration of the drug or vehicle was continued in Nijmegen, as was the way it was administered. Solutions of SLV326 were kindly donated by Solvay Pharmaceuticals, Weesp, The Netherlands.

The dosages in male and female rats were based on previously conducted shorter-term studies with SLV326, where stereotypic scratching behavior, resulting in skin wounds was noted at 3 mg/kg and above in females and 10 mg/kg in male rats. Steady-state plasma levels of SLV326 were roughly 2-fold higher in female rats. For that reason, dosages selected were 2 mg/kg for female rats, and 3 mg/kg for male rats (personal communication).

### Surgery

Animals were surgically provided with two tripolar EEG electrodes (Plastics One MS-332/2-A) under complete isoflurane anaesthesia. An incision was made to expose the skull surface. With aid of stereotactic equipment, the positions of the electrodes were determined (in mm from bregma, anterior, lateral, depth): frontal cortex: +2; -2; -1; thalamus: -2.6; -2.7; -7.3; hippocampus: -4.2; -3.6; -4.1; and brain stem: -8.8; -1.7; -5.2. Ground and reference electrodes were placed over the cerebellum bilaterally with reference on the ipsilateral side of the recording electrodes. Holes for the electrodes and for three screws were drilled in the skull at the assigned areas. Subsequently the electrodes were fixated with dental acrylic. Animals were allowed to recover for at least two weeks.

### EEG recording

EEG signals were recorded, amplified, filtered between 1 and 100 Hz and digitalized at a sample rate of 512 Hz. A Windaq system (DATAQ Instruments, Akron, OH) was used for EEG data monitoring, acquisition, and storage on hard disk for offline analysis. EEG and video recordings were made during 24 hours, starting around 1:00 p.m. Activity levels of individual animals were monitored using Passive InfraRed (PIR) motion recording devices, connected to the analog inputs of the Windaq interface.

### Data analysis

#### Seizures

The EEG files were visually inspected for the presence of epileptiform activity. This was described qualitatively in terms of the occurrence of peaks, their amplitude and their respective frequencies. Data from the brain stem electrode were not used for this study.

Whenever a seizure was observed in the EEG, corresponding behavioral data were analyzed from the video recordings. The severity of these video observed seizures was scored using Racine’s scale [25].

The total number of seizures in the EEG per animal and the accompanying behavioral scores were reported. Barnard’s exact test [26–28] was used to compare group differences in seizure occurrence.

#### Interictal EEG

The complete EEG files were visually inspected for interictal spikes by a trained expert. According to the International Federation of the Society for Electroencephalography and Clinical Neurophysiology (IFSECN) an interictal spike is defined as a transient event that is clearly distinguishable from the background EEG, with a pointed peak at conventional screen display and a duration from 20 to less than 70 ms [29]. If these were found, they were quantified by analyzing 12 segments of 5 minutes each. The segments, distributed over the 24-hour period, were used to estimate the number of interictal spikes for the entire recording period.

#### Spectral analysis

To investigate possible effects of SLV326 on the interictal EEG, 20 minutes of EEG were selected devoid of interictal spikes or otherwise epileptiform activity for each of the 35 animals. 1 control animal was excluded due to the quality of the EEG signal. These 20 minutes consisted of 10 randomly selected (using sequences generated by www.random.org) 2 minute segments. Every section was subsequently divided into 12 segments of 10 seconds each, which were assigned to either an ‘active’ or a ‘passive’ state subset, based on visual inspection of the PIR. To ensure that the separate subsets indeed showed differences in activity levels, the sum of PIRs per subset, in arbitrary units, was subsequently expressed as a percentage of the maximum deflection (S1 Fig). Active and passive subsets did not overlap. Subsequently spectral information was calculated using the Fast Fourier Transformation analysis in BrainVision Analyzer 2.0 (Brain Products GmbH), using a Hanning window and a resolution of 0.1 Hz. The spectra were then normalized, with the total power between 1 and 100 Hz being 100%.

Instead of defining frequency bands a priori, t-profiles (i.e. a t-value for every single frequency point), were made to establish a detailed comparison of differences in the frequency domains of the SLV and the control groups. Per channel, average power per frequency was compared between the groups. This was done for both subsets, i.e. active and passive behavior.

On the basis of the t-profiles areas of interest were defined; clusters of 5 of more consecutive t-values above the threshold of t = 2.0 served as indicators of differences in total relative power. Those areas of interest were subsequently compared.

For areas of interest, total relative power was compared with IBM SPSS Statistics 19 by means of Repeated measures Generalized Linear Model analyses, with State (active, passive) and EEG Channel (cortex, hippocampus and thalamus) as within-subjects factors and Treatment as between-subjects factor.

For the theta band, peak frequency per animal was established after applying a 10 step smoothing in Graphpad Prism 5.01 (GraphPad Software, Inc., 2007) on the normalized frequency spectra. Peak frequencies were compared for treatment effects by means of an unpaired Student’s t-test.

Finally, the 2 groups were compared regarding their total amount of bodily movements, as recorded with the PIR. The amount of movement was expressed as a percentage of the possible maximum (i.e. maximum amplitude multiplied by the number of data points). Treatment and state effects were analyzed by means of a Repeated Measures Generalized Linear Model analysis with State as within-subjects factor and Treatment as between-subjects factor.

## Results

### 1. Seizures

During the 24 hours of EEG recording, epileptic activity was observed in 6 out of 24 SLV326 treated animals (25%), whereas in none of the 11 controls this activity was observed. All EEG defined epileptic activities were accompanied by simultaneously occurring clinical signs of convulsive seizures (see below). This difference in seizure incidence between the drug treated group and the control group was significant (p = 0.04, using the Barnard’s exact test). No effects of gender (3 male animals with seizures and 3 females) or history of reported seizures (4 animals with previously reported seizures versus 2 without previously reported seizures) were found.

Seizure duration varied from 26 to 200 seconds, with an average duration of 83 seconds with an SD of 47 seconds (n = 42 seizures). Per animal, the number of observed seizures, which ranged between 1 and 21, and the average seizure duration, are displayed in Table 1.

**Table 1:** Number of animals showing seizures or interictal spikes, and average seizure duration.

Animal no.	Treatment	Gender	Previously observed violent movements	# Seizures	Average seizure duration (seconds)	Interictal spikes #/hour
73	SLV	M	No	13	123	76
76	SLV	M	No	2	120	7
89	SLV	M	Yes	1	101	83
183	SLV	F	Yes	1	200	57
195	SLV	F	Yes	4	97	67
199	SLV	F	Yes	21	46	149
83	SLV	M	Yes	-	-	80
88	SLV	M	Yes	-	-	48
182	SLV	F	Yes	-	-	19
71, 72, 94, 94	SLV	M	No	-	-	-
81, 82, 97	SLV	M	Yes	-	-	-
175, 178, 185, 198, 200	SLV	F	No	-	-	-
188, 191, 194	SLV	F	Yes	-	-	-
16	Control	M	-	-	-	16
1, 10, 18, 30	Control	M	-	-	-	-
101, 104, 109, 110, 118, 128	Control	F	-	-	-	-

6 animals out of 24, all treated with SLV326, showed between 1 and 21 seizures during the 24h recording period. All these animals also showed interictal spikes. 3 more SLV treated animals showed interictal spikes as well, but did not display any seizures. In the control group 1 animal had interictal spikes, although these were not generalized, in contrast to the generalized spikes seen in the SLV326 group.

#### 1.1. EEG

**Fig 1:**
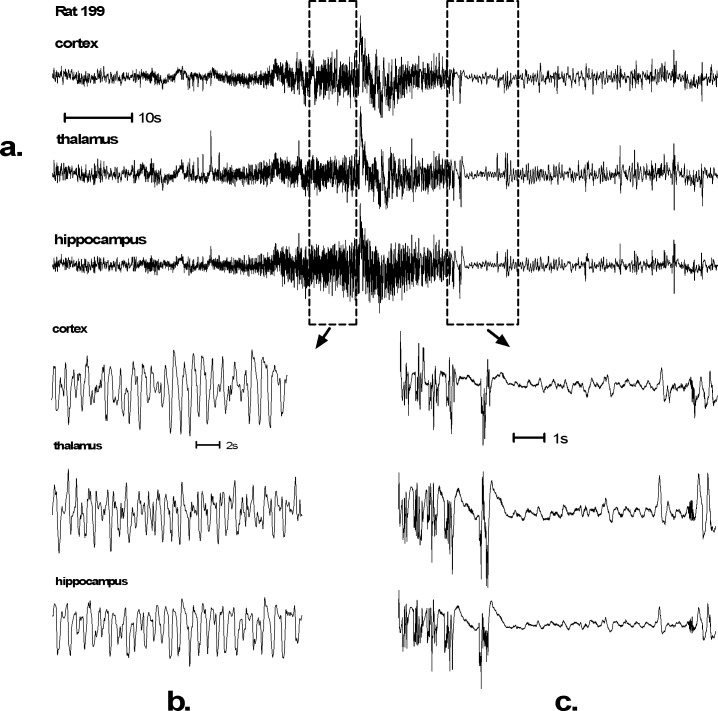
EEG recording of an SLV326 induced convulsive seizure. Such generalized seizures were found in 6 out of 24 Wistar rats treated with SLV326. Control animals were seizure free. Seizures typically start with low amplitude spikes that gradually increase in amplitude. The duration of this seizure (a.) is 30 seconds. High amplitude spike activity can be seen during the whole seizure (b.). The seizures of 3 out of 6 animals were followed by a postictal depression (c.). Behaviorally this EEG corresponds with severe tonic-clonic convulsions, Racine scale 5 (S1 File).

A typical example of seizure EEG can be seen in Fig 1. In all animals high amplitude spikes were observed simultaneously in cortex, thalamus and hippocampus (Fig 1B). Typically, these spikes are initially of low amplitude, but they increase to several times the amplitude of the background EEG (Fig 1A). In several animals a gradual waxing and waning of spike amplitude was visible, with cortex and thalamus following the same pattern. Similar waxing and waning was visible in the hippocampus.

In 3 animals postictal depressions were observed. A gradual decrease in the amplitude of the spikes was accompanied by a reduction of peak frequency that developed into a postictal depression, consisting of a smooth, virtually flat EEG. These depressions were highly variable in length and lasted between 25 and 117 seconds, before gradually returning to normal activity (Fig 1C).

#### 1.2. Behavior

Severe bilateral clonic muscle twitches of the forelimbs and the facial area and occasionally of the hind limbs were observed in these 6 animals. During the seizures, the animals displayed full rearing in a kangaroo-like posture, with the neck curled backwards and a rigid curled tail, Racine stage 5. A video of a representative seizure can be seen in the supporting materials (S1 File).

### 2. Interictal EEG

#### 2.1 Interictal spikes

In the SLV326 group, 9 of 24 animals (37.5%) showed interictal spikes in their EEG, mean 65 spikes per hour, SD 41. This included all 6 animals that showed seizures during the recording period. All interictal spikes were visible in hippocampus, thalamus and cortex simultaneously.

**Fig 2:**
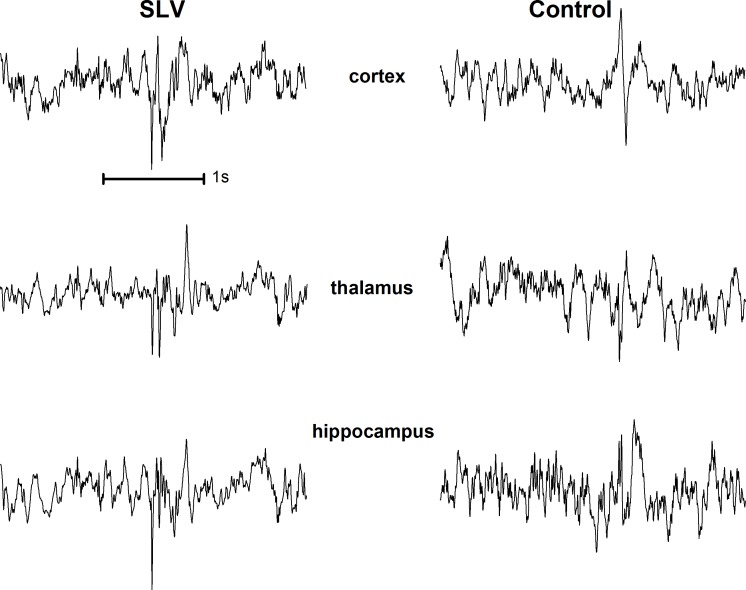
Examples of interictal spikes from a SLV326 (left) and a control animal (right). An average of 65 spikes per hour was found in 9 out of 24 SLV treated animals, versus 1 control animal out of 11 having 16 spikes per hour. Interictal spikes in the SLV group were all visible on all 3 EEG channels (left), whereas the spikes in the control animal were local, on the cortex (right).

Interictal spikes were very rare in the control group: only 1 out of 11 animals (9%) showed 16 spikes per hour. However, these spikes were only present in the cortical EEG and never present in hippocampus and thalamus. Examples of interictal spikes from both groups can be seen in Fig 2.

#### 2.2 Spectral analysis

**Fig 3:**
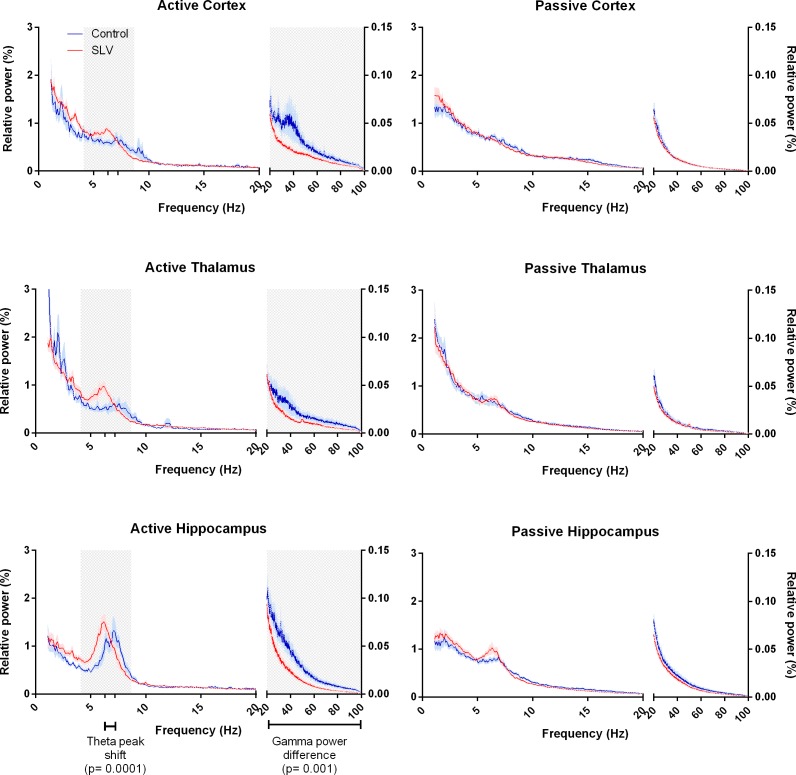
Frequency spectra of background EEG in active and passive states. Areas of interest, determined on the basis of t-profiles, are marked in grey. During activity (left side) there are clear theta peaks in SLV326 treated animals as well as controls, with a significantly lower frequency of the theta peak compared to that of the controls. Also, a decrease in relative gamma power is noticeable during activity. No such differences are present in the passive spectra.

The EEG segments of 34 animals (10 controls and 24 SLV326) used for spectral analysis were selected by means of visual inspection of the PIR; the quantified movement of passive epochs that were used did not differ between the control (14.8% (SD 4.8)) and SLV treated rats (16.0% (SD 4.0)). In the active condition the quantified movement was 41.8% (SD 12.2) for the control group and 43.6% (SD 8.8) for SLV treated rats and again this did not differ. Normalized frequency spectra of the EEG are shown in Fig 3. T-profiles were constructed for the normalized spectra of the EEG segments. The frequency spectra of passive behavior are characterized by a relatively large amount of delta power. No effects of SLV326 were found in the t-profile of the passive condition. Both the SLV326 and control group showed similar passive frequency spectra, with no distinct theta peak.

**Fig 4:**
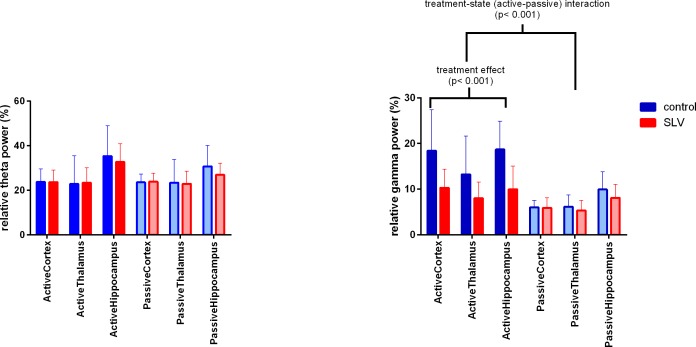
Total relative power for the theta (left) and gamma (right) areas of interest. No differences in theta power were observed, 27.9% (SD 8.62) for SLV versus 24.7% (SD 6.87) for controls. There was however a significant treatment-state interaction in the gamma range, (F(1,33) = 22.9, p< 0.001). In the active state a treatment effect was observed, as total relative power in SLV treated is significantly lower (p < .05) than in control animals, 9.45% (SD 4.25), compared to 16.8% (SD 7.86). No such shift in relative power was found in the passive state. Mean and SD are given.

During (locomotor) activity, all animals show a clear theta peak in their frequency spectra, which was most distinct in the hippocampal channel. Areas of interest, observed in the t-profile, were found in the theta (4.1–8.7 Hz) and the gamma (23.4–99.9 Hz) range. SLV326 treated animals had a lower theta peak frequency, 6.3 Hz (SD 0.51), compared to 7.2 Hz (SD 0.53) in controls (t(28) = 4.390, p < 0.001). However, as can be seen in Fig 4, there was no difference in theta power, 27.9% (SD 8.62) for SLV versus 24.7% (SD 6.87) for controls. A treatment-state interaction was found in gamma power (F(1,33) = 22.9, p< 0.001). Post hoc analysis showed that during active behavior the power was lower (p < .05) in the SLV group, 9.45% (SD 4.25), compared to 16.8% (SD 7.86) in the control group. SLV326 did not differ from the control in the passive condition 6.45% (SD 2.46) for SLV versus 7.36% (SD 2.67) for controls.

## Discussion

Our combined EEG-behavioral study with Wistar rats chronically treated with the CB1 antagonist SLV326 showed two major findings. Firstly, the behavioral spasms, which were observed during daily clinical observations, were identified as generalized convulsive seizures. The aberrant activity in the EEG showing generalized, high amplitude sharp spiky activity, and in some cases post-ictal depression, was concomitantly accompanied by severe clinical symptoms. Therefore it can be concluded that these muscle spasms are indeed epileptic phenomena.

The second major finding was that spectral changes were found, specifically during active behavior, suggesting that locomotor activity or sensory motor processing may be affected.

The convulsive seizures were present in 6 out of 24 treated animals (25%). All seizures were scored 4 or 5 on Racine’s scale. The violent movements (or muscle spasms as the biotechnicians really described it like that) were rhythmic movements which developed in time and represented well-ordered consequence of motor manifestations. The complex behavioral pattern is known to be specific for epileptic seizures generated by limbic structures in rodents.

Additionally, interictal spikes were found in more SLV treated animals (37.5%). Interictal spikes can serve as a sensitive marker for epilepsy [22, 30] or epileptogenesis [31, 32]. Therefore it is likely that the number of animals with seizures is actually larger than 25%. The spikes found in the treated animals were all generalized, whereas the spikes in the single control animal were localized. Epileptic spikes are expected to be found in close proximity to the epileptic focus. This might indicate the spikes in the control animal to be local cortical changes due to electrode. In view of the limbic nature of the generalized SLV326 seizures, a cortical focus is not likely, but cortical electrodes can register high limbic activity. However, much is still unclear about the relationship between seizures and spikes [33], and conflicting results have been described in literature [34].

The presumption that more animals than the 25% we observed might have seizures is also dictated by behavioral observations made during the regulatory repeated dose study that preceded the present EEG study. The animals’ behavior was monitored during daily cage observations; aberrant behavior, described as muscle spasms, was observed in 70% of the animals of the treatment group (totally 39 animals out of 60) during the course of the treatment period (personal communication). Given the short period of EEG recordings (24 hours) in the present study, it is likely that seizures would be observed in more animals over a longer time frame. Moreover, the animals with EEG-behavioral seizures consisted of an equal amount of animals with and without previously reported “spasms”. We expect that the actual number of animals with seizures might be higher than 25%, or even 70%.

The ‘hands-off’ approach during the EEG recordings excludes the possibility that external influences, such as handling stress, were culprit, as was suggested for rimonabant by the European Medicines Agency; the seizures that we noticed did occur in their home cages and occurred spontaneously. The animals were freely moving and were habituated and adapted to the EEG leads and EEG recording home cages. Moreover, the seizures were evenly distributed over the 24 hours of recording, and no relation with the time of gavage was found. Therefore, handling can be excluded as a factor.

This study confirms the findings of our previous study, using the CB1 antagonist rimonabant [6]; this antagonist induced de novo limbic seizures in healthy Wistar rats as well. These findings suggest that the EEG changes that were observed in both studies are related to CB1 antagonism, rather than being compound-specific. Data from Vinogradova, Shatskova [35], showed that CB1 antagonists augment the spreading of brainstem seizures, induced by audiogenic stimulation, to limbic areas, signifying that a proper functioning of the endocannabinoid system in limbic areas is of importance in the protection against seizures.

Results from animal models for brain injuries suggest a seizure protective role of the endocannabinoid system as well. In a model of chronic brain injury, viral encephalopathy, rimonabant induced spontaneous seizures [36]. Also, rimonabant increases the severity of pilocarpine-induced seizures [16, 37]. Moreover, impairment of endocannabinoid synthesis increased the seizure susceptibility [38]. G protein Gq/G11 knockout mice, which have impaired endocannabinoid synthesis, showed spontaneous epileptic seizures. It is notable that both the frequency of seizures in each animal and the number of affected animals increased with age [38].

In all, the present study strengthens our previous proposal that the endogenous cannabinoid system protects against seizures. Indeed, cocaine-induced seizures in mice were inhibited by enhancing the endocannabinoid system [39]. These inhibitory effects were not observed in the presence of a CB1 antagonist.

Since the limbic system is especially vulnerable to injuries [40], it is not surprising that limbic seizures were observed in our animals, including hippocampal seizure activity. In humans, age-dependent seizures are often of a limbic nature [41, 42]. Apart from the epileptic activity induced by SLV326, changes in spectral content of background EEG were observed. When active, SLV326 treated animals display a lowering of the theta peak frequency and a decrease in relative gamma power. Because the spectra were normalized, a decrease in gamma power coincides with an increase of the power in other (low) frequency bands.

It is interesting to speculate what this means in terms of changes in the absolute spectrum. Our results suggest that CB1 antagonists induce a reduction of gamma power rather than an increase in the lower end of the frequency spectrum. Indeed, the absolute power spectra of our data (not shown) seem to confirm this, although no significant differences were found due to the high variance between animals.

So far only reports on the effects of agonists on the spectral power have been published. In humans a CB1 agonist causes a decrease in the EEG power spectrum between 7 and 27 Hz [23]. Effects of cannabinoids on the gamma band have been described in animals [24] as well as in humans. Nottage, Stone [43] report a THC-induced increase in the magnitude of gamma activity. The decrease in gamma activity we found, after exposure to an antagonist, is in line with this. However, CB1 agonists have also been found to reduce gamma power [44], although in this case repeated exposure to cannabis blunted the response. When taking into consideration that the gamma band is typically associated with higher cognitive functions, it is not surprising that the complete picture is more complex. Indeed, effects of cannabis on gamma activity have been found to be dependent on dose [45] and time after administration [43]. Interestingly, the effects on the EEG spectrum were state-dependent, as they were only present when the animals were highly active. Indeed, theta rhythms are associated with locomotor activity [46]. Nevertheless, we did not find any changes in the quantity of locomotor behavior (S1 Fig; p = 0.5). Theta rhythm is shown to be a marker of complex behavior [47]. It is likely that the behavioral effects caused by SLV326 are of a more complex nature than, and therefore do not show up in the PIR, as CB1 receptors are known to be involved in, among others, compulsion and food intake behavior, all of which can be observed predominantly during active wakefulness [48]. Sprague-Dawley rats treated with CB1 antagonist AM-251 during adolescence showed more active stress coping behavior and greater risk assessment during adulthood, but no changes in locomotor activity [49]. Moreover, in several studies using animal models for cognitive impairment CB1 antagonists have been shown to improve cognition [50–54].

Whether and how the epilepsy and the spectral changes are connected remains to be investigated. In humans with epilepsy both differences in absolute and relative power of the frequency spectrum have been found [55]. In children, epilepsy was found to be highly correlated with changes in the alpha band [56]. A study from Maheshwari, Marks [57] suggests that relative gamma power may serve as a biomarker of AED efficacy in absence epilepsy; drugs that reduce interictal gamma power increase the incidence of absence seizures. This might imply that the observed deficit in gamma power in SLV-treated rats can be ascribed to enhanced susceptibility to seizures.

In summary, blocking of the endogenous cannabinoid system makes the healthy rat brain more vulnerable to epilepsy. Additionally, it alters the frequency spectrum of the EEG when rats are highly active, suggesting effects on complex sensory motor functions.

The role of the endocannabinoid system is still far from clear, but its properties make it very suited for protection from excessive oscillatory activity. A better understanding of the epileptogenesis induced by removing or blocking this protection system may contribute to a better understanding of the endogenous cannabinoid system altogether.

## Supporting Information

S1 Fig Total sums of PIR. To ensure that allocation to ‘active’ and ‘passive’ subsets based on visual inspection of the Passive Infrared (PIR) motion detection signal was performed successfully, the sums of all PIR signals per animal were expressed as a percentage of the maximum possible deflection of the PIR. For the ‘active’ subset, this was 41.8% SEM 3.98 for controls and 43.6% SEM 1.79 for SLV treated animals. For the ‘passive’ subset, this was 14.8% SEM 1.52 for controls and 16.0% SEM 0.80 for SLV-treated animals. The PIR sums were found be significantly different for state (active, passive; F (1, 32) = 246.5; p < 0,0001). No treatment effect was found, F (1, 32) = 0.4589; p = 0.5, nor was there a state-treatment interaction, F (1, 32) = 0.02019; p = 0.9.
(TIF)

S1 File Video recording of a seizure during the EEG study. In this video a representative seizure can be seen. Note the rigid tail and kangaroo pose of the animal, as well as the clonus of the forelimbs.
(ZIP)
