# Supplementary figures and images for: Cannabinoid antagonist SLV326 induces convulsive seizures and changes in the interictal EEG in rats
Source: PLoS One. 2017 Feb 2;12(2):e0165363. doi: 10.1371/journal.pone.0165363 (PMC5289424; doi:10.1371/journal.pone.0165363)

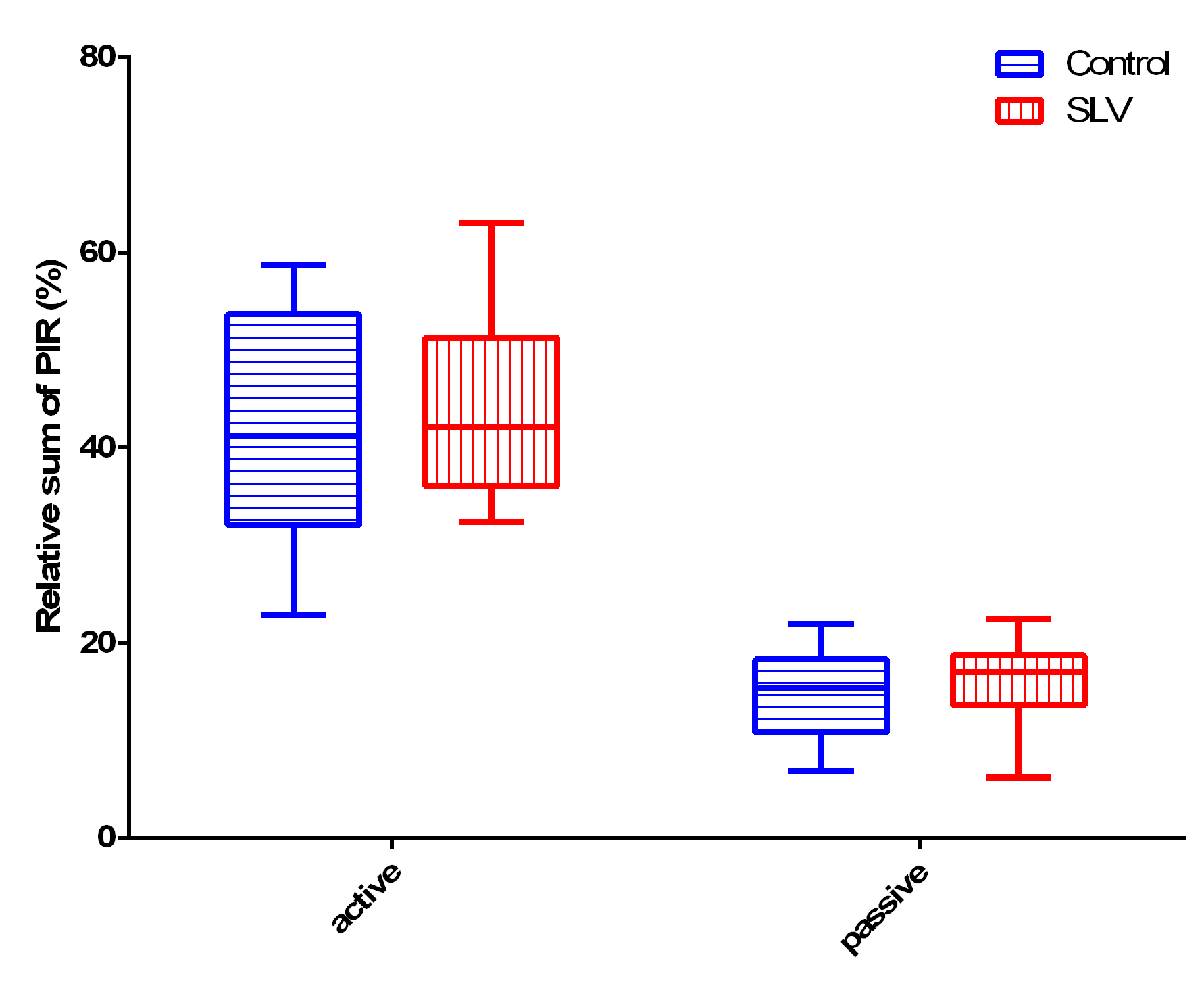

Supplement: S1 Fig — To ensure that allocation to ‘active’ and ‘passive’ subsets based on visual inspection of the Passive Infrared (PIR) motion detection signal was performed successfully, the sums of all PIR signals per animal were expressed as a percentage of the maximum possible deflection of the PIR. For the ‘active’ subset, this was 41.8% SEM 3.98 for controls and 43.6% SEM 1.79 for SLV treated animals. For the ‘passive’ subset, this was 14.8% SEM 1.52 for controls and 16.0% SEM 0.80 for SLV-treated animals. The PIR sums were found be significantly different for state (active, passive; F (1, 32) = 246.5; p < 0,0001). No treatment effect was found, F (1, 32) = 0.4589; p = 0.5, nor was there a state-treatment interaction, F (1, 32) = 0.02019; p = 0.9. (TIF) [file pone.0165363.s001.tif]
